# Altruism and the pressure to share: Lab evidence from Tanzania

**DOI:** 10.1371/journal.pone.0212747

**Published:** 2019-05-21

**Authors:** Salvatore Di Falco, Razack Lokina, Peter Martinsson, Paolo Pin

**Affiliations:** 1 Institute of Economics and Econometrics, Geneva School of Economics and Management, University of Geneva, Geneva, Switzerland; 2 Department of Economics, University of Dar Es Salaam, Dar Es Salaam, Tanzania; 3 Department of Economics, University of Gothenburg, Gothenburg, Sweden; 4 Dipartimento di Economia Politica e Statistica, Università di Siena, Siena, Italy; 5 IGIER and BIDSA, Università Bocconi, Milano, Italy; Universidad Loyola Andalucia, SPAIN

## Abstract

We propose a novel laboratory experiment to document the pressure to share income within social networks in Africa. We find that the redistributive pressure exerted via the possibility of receiving a claim increases altruism, while the possibility of hiding from such claim reduces it. Our results indicate that sharing norms are crucial drivers of giving to other members of the network. We also find that pressure to share has a detrimental effect on the undertaking of profitable but risky investments.

## 1. Introduction

The sharing of resources within social networks is a strong norm in many developing countries. Cash, goods and in kind services are transferred to other members either voluntarily or as a response to a direct request [1–9]. This behavior can be thought to be an expression of altruistic preferences towards relatives and nonrelatives [10–12]. An important issue is whether altruism is instead the product of binding sharing norms that may have potentially negative economic implications both in terms of less investment in income generating activities and an increase in resources spent on hiding income. As noted by [13] (p.192), the rich may help the poor ‘less from a spirit of liberality than as response to the palpable pressure their neighbours and kin brought to bear upon them.’ A recent and growing body of literature, using both observational and experimental approaches, provides empirical evidence that this may be the case. It shows, indeed, that individuals may implement costly activities such as hiding to prevent redistributive pressure [14–20].

In this paper we use a laboratory set up to provide experimental evidence showing how *explicit* redistributive pressure affects both an individual’s decision to share resources and to undertake a profitable investment. The laboratory environment is particularly useful in addressing these research questions (see [21] for a comprehensive review). First, the random allocation of individuals to different conditions allows for the estimation of effects that are not correlated with other variables. Findings are therefore not prone to omitted variables bias. Second, the mechanisms underlying a specific empirical relation can be further studied and probed. We, thus, provide an experimental design where sharing pressure and the possibility to hide from it can be manipulated. We then observe both the subjects’ investment decisions and their redistribution of resources within their social network.

The experiment was conducted in rural Tanzania and it was framed in the context of a typical social network in a developing country. We used a modified dictator game with multiple recipients where there is an important interplay between investment decisions by the senders, explicit claims from the recipients and possibility to hide at a cost. In some treatments, recipients are thus allowed to claim some of the resources from the senders they are related to, while in others the senders had the possibility to hide their resources at a cost. This is in the spirit of [22–23]. Both experiments indeed allow for the possibility to opt out from a situation of giving. This experimental condition allows us to explicitly study the role of pressure on investment and resource sharing. This is a novel experimental feature that allows to explicitly and simultaneously testing behavioral responses both in terms of investment and giving decisions under redistributive pressure.

### Context of social ties

Framing and creating artificial social ties in the laboratory is used in studies on group identities and individual decisions (e.g., [24–25]). This is a very central social organization and key provider of social capital in Sub Saharan Africa. Differently from other types of networks (e.g., friendships), membership is determined via bloodlines or marriage and it is not “the result of individual choice” [26]. Ties created by bloodlines promote altruism and regulate access to resources and services (e.g. [27–28]) as well as govern social relationships and marital customs. Redistribution and sharing of resources within social network is a means to provide economic and social security ([1] and [29]). In the absence of formal insurance and credit markets, these networks provide opportunities for risk sharing and a social safety net for the unlucky ([3], [5], [9] and [30–31]). This type of social networks may also matter because “the ties of common experience, altruism and heritage among family members enable families to transcend some of the information problems barring the development of impersonal markets” ([32], p.1167). [33] extend the basic mutual insurance under imperfect commitment model, and consider the implications of altruism entering into sharing relations. Altruism tends to ameliorate commitment problems, and increases the potential gains from income pooling and mutual insurance ([34]). Members of the social network may thus claim assistance from others when necessary. Networks may also help to restrain opportunistic behavior of members or free riding, lower transaction costs, facilitate the exchange of information, and enable communities to overcome social dilemma situations.

Emphasizing the strength of the moral obligations towards the less lucky members of the network, [35] refer to the concept of “forced solidarity”. They show that, in Cameroon, a significant portion of sample individuals borrowed money at a cost even if they had available savings. They argue that the reason for this costly behavior was to avoid the pressure from others to share their resources by using borrowing to signal financial difficulties. Social sanctions (e.g., social stigmas) may be faced by those who defect from the moral imperative of sharing ([36]). [2], for instance, discusses the role of witchcraft and ostracism. These are very important sanctioning mechanisms that make it unlikely for individuals to avoid the sharing requests of the network ([37]). Social stigma, as well as implications of any retaliation, can “fall on the defectors as well as on other members of their clan, increasing the cost of breaching the contract” ([38], p.1733).

### Findings

Our main results are as follows. First, we find that sharing pressure significantly increases altruistic behavior. The highest level of giving is, indeed, found when subjects are exposed to explicit sharing pressure from perfectly informed members of their social network (interestingly, 96% of the receivers in the *claim* treatment made a claim to others). This result is consistent with existing findings, from both developed and developing countries, interpreting hiding as a strategy to reduce giving e.g. redistributing a smaller fraction of resources ([18–19, 22–23, 39–40]). Second, we find evidence that the size of investments are smaller when the redistributive pressure is larger and that individuals use hiding (at a cost) to fend off requests from other members of the network. Third, we find that income affects giving decisions only when redistributive pressure is higher. This suggests that giving behavior is based also on social norms and it may not only be driven by altruistic preferences ([10, 41–42]).

These results are relevant for three broad strands of literature. First, and most obvious, is the literature on altruism and giving ([10–12, 43–45]). Our study provides a crucial alternative explanation of sharing resources. The act of giving (implying resource sharing) can be a reflection of pure altruistic preferences, or it can be the result of strong sharing norms that might be amplified by pressure from others. This has been termed in the behavioral literature ‘involuntary giving’ ([23, 28, 46]). Using the laboratory environment, we find that a non-negligible part of sharing is the results of social norms and sense of duty. Second, our study relates to the strand of literature on the economic implications of social networks. Households anticipating that their income will be subject to redistributive pressure may try to avoid this by making ill-suited economic decisions. They may, for instance, change their consumption, saving and investment decisions ([15, 47–48]). [18] provided some experimental evidence showing that females are less likely to undertake profitable, but risky prospects when observed by their relatives. [19] showed that sharing pressure reduces the productive incentives of entrepreneurs, leading them to invest less in their business than they otherwise would. [49] also showed that individuals belonging to larger social networks are less likely to adopt some agricultural technologies in rural Ethiopia. In most of these papers, avoiding the pressure to share is the key explanation behind the observed pattern in the outcome variables. In this literature sharing pressure is inferred by the simple comparison of a situation in which one’s resources or investments are observable to the others, versus a situation where hiding them is possible. Our experimental design aims to probe this mechanism further by explicitly relating claims to hiding and investment decisions. Third, our paper relates to the small body of literature on income hiding in village economies. Hiding income can be an explanation for incomplete risk sharing and therefore may play a very important role in explaining barriers to insurance in a developing country context ([17, 19]). It also plays a role in the possibility to achieve first best allocation in dynamic consumption models ([50]). To our knowledge, this is the first paper providing the laboratory evidence of the hiding income hypothesis. Our main novelty lies in the fact that we include explicitly peers in the experimental setup, and we allow them to make explicit claims. We propose also a hypothesis based on rational inattention that is consistent with what we find in the experiment.

This paper proceeds as follows. Section 2 presents our experimental design and general procedures. Section 3 details experimental results. Section 4 investigates the nature of the norms followed by subjects with a simple theoretical approach. Section 5 concludes the paper.

## 2. Experimental design and procedures

The key feature of our experimental design is to replicate a real life situation, typical in context of a Sub Saharan Africa, where people can make a risky investment decision and they potentially face claims to share their resources with others. We, therefore, created fictitious social networks from groups of 6 people with different artificial ties, as described in Table 1. There are 2 types of individuals in the network: type A subjects (senders) and type B subjects (receivers). Subjects are presented with hypothetical links characterized by different degrees of genetic distance. The relationship between type A and B thus varies artificially in the degree of closeness. Every subject in a group is therefore interacting with what corresponds in the framing of the experiment to a close family member, a member of the extended family or a village neighbor. This structure can be described as a ring structure where links partially overlap. So, for instance, B2 is close member of the family of A2, member of the extended family of A1 and neighbor of A3.

**Table 1:** Social network in the experiment.

	Subject B1	Subject B2	Subject B3
Subject A1	Close family	Neighbor	Extended family
Subject A2	Extended family	Close family	Neighbor
Subject A3	Neighbor	Extended family	Close family

Different relationships represent different strength of ties. Different strength of ties implies different levels of altruism and sharing ([51]). To capture this feature we differentiate the maximal amount of giving according to different strength of social ties. Subjects A can therefore freely give a maximum of 50% of their money to a member of the close family (e.g. siblings), a maximum of 30% to a member of her extended family, and a maximum of 20% to a neighbor. This bounding structure, while allows participants to freely allocate their resources to the others, does emphasizes the fact that the closeness of a relationship is important ([11–12]). Findings from dictator games also show that as social relationships become more distant less resource are given (e.g., [52–53]). This is also consistent with the notion of altruism towards relatives in economics ([28, 51, 54]), evolutionary biology and psychology. Individuals will preferentially assist their close relatives ([27, 29, 55]). Help and cooperation is also more likely to happen among relatives rather than non-relatives ([56]).

Each subject has access to the same structured social network. They were randomly assigned into one of 4 conditions (described below) as well as to the role of being type A or type B subjects. Conditions are all characterized by anonymity and no real-life elements were present in the analysis. Subjects could not see with whom they were matched with or what role subjects in other rooms were assigned. A chart of the structure of the network was displayed in the rooms in order to facilitate the understanding of the matching process.

The experiment is played in one shot. Type A subjects are endowed with 5000 ECU (Experimental Currency Units) corresponding to 5000 Tanzanian Shillings, while type B subjects are endowed with 2000 ECU. The daily wage for a participant in the region is 7000 shillings a day. The first decision that type A subjects have to make is how much of the endowment to invest in a risky project. If the project is successful, the amount invested will be tripled. If it is instead unsuccessful there is no return and the investment is lost. The type A subjects can invest how much they want of they own endowment (from 0 to 5000 ECU). The probability of a successful outcome is 50%. Once we have determined the outcome of the investment by tossing a coin, the type A subject decide how much she would like to give of her resources to the three type B subjects who are related to her (her social network). We will observe this giving decision under a number of different experimental conditions. There are two key parameters in our design: (i) claim–whether or not the type B subject can claim money from the type A subjects in her network; and, (ii) hide–whether or not the type A subject can hide her income at a cost following a positive outcome from the risky investment. It should be stressed that no deception was in place. The set of instructions given to the B players in fact mentioned that the A players would have the possibility to hide at a cost. For example, in the claim and hide condition a B player who was informed that the A player paired with her had 5000 ECU could infer that either the latter did not invest or could have decided to hide resources. The 2x2 design generates 4 conditions in total: one control and three treatments. In the baseline (control) condition, the type B subjects are assigned a passive role. In essence, this is a standard dictator game (with multiple receivers and senders) and it is used to measure pro-sociality without any external pressure from members of their network.

The pressure for sharing is explicit in the *claim* experimental condition (or treatment). In this condition, the type B subjects are able to make a claim on resources from any type A subject who is a member of their network. This treatment captures the extent of network redistributive pressure. Claims are not binding by design. Type A subjects can give zero if they wish. Again, we impose different (and consistent with the previous) bounds to distinguish and characterize the strength of the ties. The maximum amount that this type B subject can claim is 50% from a member of her close family, 30% from extended family and 20% from a neighbor, consistent with the type A subject’s giving constraints. This implies that both sharing and hiding are affected by genetic distance in the same way. Subjects A potentially share more with closer kins an face higher costs when they hide from their closer relative. This prevents possible strategic behavior as one player could in principle condition the choice on hiding on the outcome of the lottery. The way the social ties bind is the same. All type B subjects linked to a type A subject will know her total income after the risky decision is made. To allow for a possible evasive response from the sharing pressure we introduced a *hide* treatment. In this treatment the type A subject can hide her positive income from the investment decision. This comes at a different cost: 500 ECU to hide from close family, 300 ECU to hide from extended family and 200 ECU to hide earnings from a neighbor. It is, therefore, morally more costly to hide from closely related individuals rather than more distant ones. Obviously, type A subject must make this decision before the outcome of the risky investment is known. These two choices can be thought as simultaneous. If the type A subject decides to hide the outcomes of the investment decision, the type B subject will be informed that the outcome equals the initial endowment (i.e. 5000 ECU) even if they made a successful investment. In the event of a loss, the (true) income of the type A subject after the loss is revealed by type B subjects (i.e. 5000 ECU minus the invested amount). This replicates the real-life situation where the initial investment has been hidden, but no effort is taken to hide the loss at the time the loss is realized.

In a last treatment called *hide and claim*, while type A subjects can hide their investment outcome, type B subjects can still make a claim. This condition assesses if hiding at a cost is a viable strategy to buffer against the explicit sharing pressure exerted by the network. We summarize our experimental design in Tables 2 and 3. It can be seen how the design allows us to investigate both separate and joint effect of hiding and claims on investment decisions as well as on sharing behavior.

**Table 2:** Experimental design.

	Give without claims	Give in response to claims
Hiding income not possible	Baseline	Claim
Hiding income is possible	Hide	Hide and claim

**Table 3:** Resources sent to network members (t test, difference in means).

	No claims	Claims	P-values
Hiding income not possible	1093	1806	0.09
Hiding income is possible	418	588	0.48
P-values	0.01	0.01	

**Fig 1:**
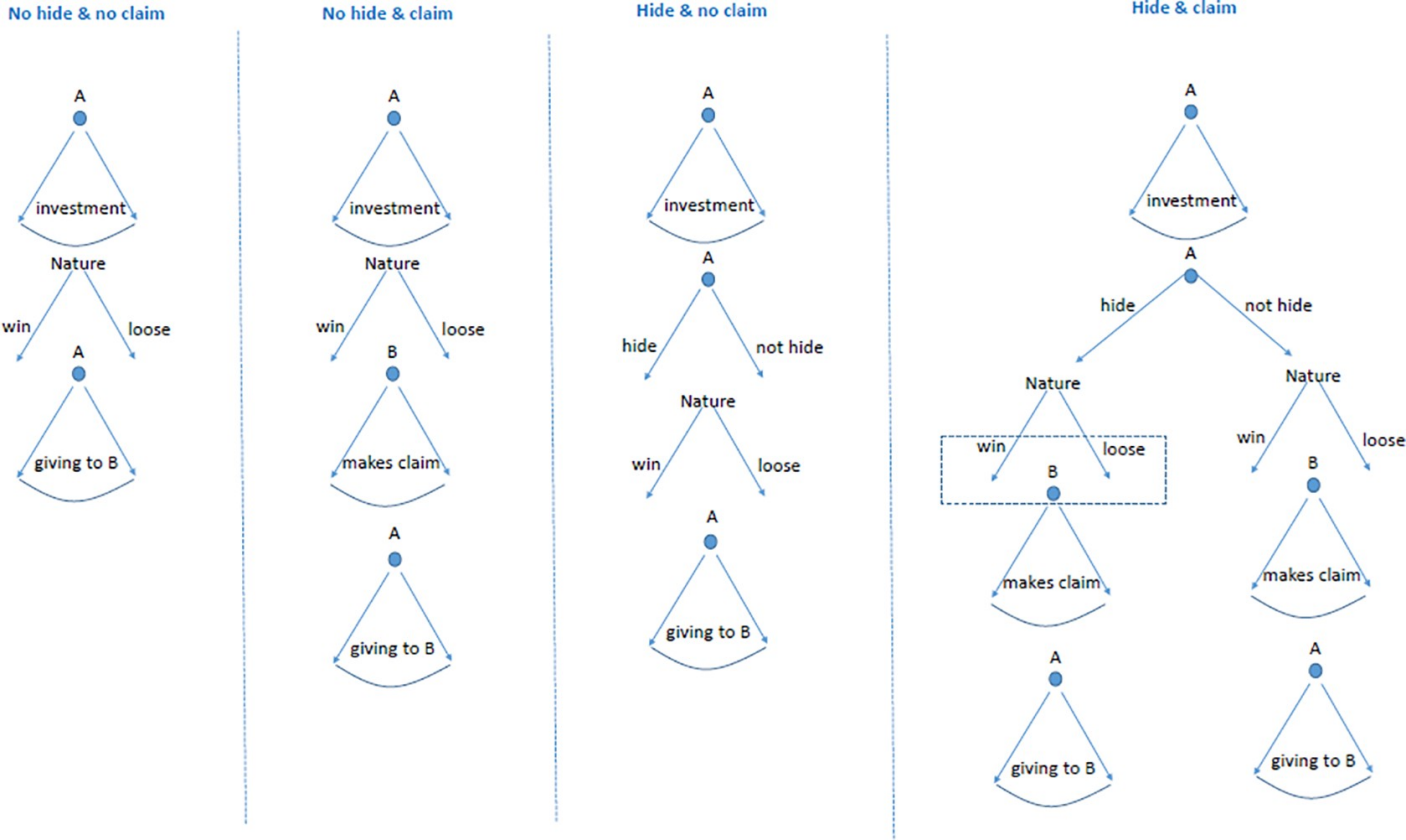
Game theoretical representation of the four experimental conditions. Note first that from a game theoretical perspective the moves of B are irrelevant information for A, even if she has other regarding preferences (i.e., this is just ‘cheap talk’). So, from a theoretical perspective, these four games in extensive form are equivalent, however we define payoffs. Note also that B moves only in the ‘claim’ treatments, but her information set is different in the two cases. In the ‘no hide & claim’ case, B has perfect knowledge of the history of the game when she makes the claim. In the ‘hide & claim’ case, when B has to move, she faces uncertainty if A has chosen to hide (in the left arm), and in that position (in that information set) she also does not know the investment that A made.

In the *hide and claim* treatment, type A subjects make the decision to hide their final income from any or all the type B subjects related to them before any investment is made. They then decide how much (if any) of their initial resources to invest in a risky prospect and how much to save. Following the outcome, if the type A subject has not chosen to hide the outcome, their final income is communicated to the type B subjects (in the no hiding treatment). In the case of the type A subject deciding to hide the outcome, the type B subjects will be told that the type A subject has 5000 ECU in the case of a successful investment, or the actual amount remaining after investment in the case of failure. The costs of hiding and the maximum giving and claim amounts remain as in other treatments. The complete game theoretical representation of conditions is presented in Fig 1.

### Procedures

A total of 240 undergraduate students were recruited at the Sokoine University of Agriculture (SUA) in Morogoro, Tanzania, which is the second largest university in Tanzania. The university gives courses in a wide range of subjects such as agriculture, business, development, geography and planning. Students participated voluntarily in response to advertising for a paid decision-making experiment. The IRB was not needed as the study was not of medical nature. The treatments are not drug or placebo. No physical or psychological risk is entailed. Participants gave their informed consent to participate at the beginning of the experiment by ticking a box on the consent form to express consent. The experiments were always played in condition of anonymity. Upon arriving at the experiment location, participants were randomly allocated to different room representing different roles. When the experiment began, they received oral and written instructions in both Swahili and English. A short quiz was then distributed to ensure that the tasks were fully understood. The correct answers were written subsequently on the black board. Participants then had to possibility to ask any remaining questions they had in privacy. Each subject would share the room with others playing the same role as them. They could not therefore see with whom they were paired with. All answers and decision taken in the experiments were private and could not be observed by others. In the room, subjects were given additional time to read the instructions and they were then asked to fill a small questionnaire to collect some basic socio-economic data. Payments were made at the end of the experiment in the respective rooms. Each room was vacated in a sequential fashion to avoid that participants with different roles could exit the building at the same time. Given that we address an investment decision, risk preferences may matter. We therefore conducted a very simple elicitation task. Participant were asked to self -assess their risk tolerance on a scale 1 to 6. We then used this simple hypothetical measure as a control in the regressions (see, [57] for the validity of hypothetical measures and [58], for the mapping of elicited risk preferences in real life in developing countries). The full list of variables and their summary is reported in S1 Table. The balance test, reported in S2 Table, shows some differences in some controls variables. It should be noted, though, that only the variable married is, in fact, statistically significant across the different treatments. While the differences do not seem suggestive of any systematic pattern, they clearly highlight a situation that is not ideal.

## 3. Results

In this section we present the results of the set of the experiments. We first present the simple t tests (Table 3), and then we present the econometric models of the giving analysis and the results of the investment behavior. We find that subjects that faced redistributive pressure increased giving behavior. Sharing pressure plays a significant role in determining the amount of resources that will be distributed within the network. Type A subjects send the largest amount (1806 ECU about 26% of the average experimental endowment) to the type B subjects if they are exposed to explicit claims and cannot hide their income at a cost (in the *claim no hide* experimental condition). A polar opposite situation is presented in the *hide and no claim* treatment. In this condition type B subjects are not able to make claims; thus type A subjects can further reduce sharing by the possibility of hiding their resources. In the *hide and no claim* treatment we find that the lowest amount of resource is sent, on average, by type A subjects (418 ECU about 7% of their experimental endowment) to type B subjects. The experimental condition that does not allow individuals to be exposed to redistributive pressure (claims) is the standard dictator game. In this set up senders also do not have the possibility to hide at a cost. The amount of resources sent to the network is 1093 ECU (15% of the experimental endowment). Of special interest is the experimental condition where we can simultaneously observe redistributive pressure and the possibility to escape from it. That is, the *claim and hide* treatment. We test, therefore, how much giving in response to claims is sensitive to the possibility of hiding. We find that in the *claim and hide* treatment, type A subject send 588 ECU in response to the claims of the type B subject. This corresponds to only 8% of the average experimental income. A figure that stands in stark contrasts with the 26% found in the absence of the possibility to hide at a cost.

We also run a regression model (reported in Table 4) to test the impact of the different treatments on the amount sent from type A subjects to the type B subjects while adding a large set of controls such as age, gender, religion, land owned by family, if help their parents in farming activities, year of study, marital status, and elicited risk preferences. Results, reported in column (1), are very consistent with the simple differences in means.

**Table 4:** Resources sent to network members. OLS estimates.

Baseline: Standard dictator game (no claims, no hiding)		
	Controls	Controls and claims
	(1)	(2)
Claims	566.5***	1075.7***
	(85.27)	(228.1)
Hiding and claims	-957.3***	0
	(163.1)	(0.00000284)
Hiding and no claims	-642.9***	0
	(102.2)	(1.16e-13)
Age	-18.48	-34.20
	(23.62)	(37.53)
Gender	-25.99	181.8***
	(235.9)	(48.93)
Religion	8.388	-705.0***
	(300.1)	(197.3)
Help parents	300.5*	245.5***
	(160.0)	(64.05)
Land	0.779***	-4.797
	(0.0594)	(5.897)
Risk aversion	53.49	66.92***
	(71.08)	(11.84)
Experimental endowment	0.126	0.155***
	(0.0789)	(0.00760)
Married	710.2***	406.4***
	(273.4)	(14.00)
Claims received		0.0935
		(0.163)
*N*	120	60
adj. *R*^2^	0.386	0.541

Session clustered standard errors in parentheses* *p* < 0.10, ** *p* < 0.05, *** *p* < 0.01. Small cluster correction applied ([60]). All specifications include year of study fixed effects. Constants not reported.

The omitted category is the baseline scenario (standard dictator game: no claims and no hiding). Results show that the presence of an explicit claim from one’s social network increases giving by the senders while the experimental condition as the opposite effect of reducing it. Column (2) reports the estimation results with the inclusion of the total amount of claims received by the senders as a control. This is a very relevant variable as it allows to control for the extent of the sharing pressure. Its estimated coefficient is positive and highly statistically significant. Senders increase their transfers when facing larger requests.

Our design also allows us to test if hypothetical genetic distance matters in the way claims are dealt with. We test if senders are giving more in response to claims that are placed by other participants that are presented to them as closer relatives. We thus test more resources are sent to the members of the social network with whom there are hypothetical closer links. To take into consideration for the simultaneity of the decisions taken by the sender a Seemingly Unrelated Regression (SUR) model is adopted ([59]). We therefore estimate the three models simultaneously and allow for the correlation in the error terms. Table 5 shows that results are qualitatively consistent with the simple testing of difference in means. Standard errors were clustered at the session level. Correction for small number of clusters was applied ([60]). The omitted reference group is the *hiding and no claim* treatment. We make this choice because this is the benchmark case where individuals are exposed to the lower levels of peer pressure from the network. We add a set of controls in column (1) and column (2). In the latter we present the results where we control for the amount of the claims presented by the member of the close family, the extended family and the neighbor respectively.

**Table 5:** Total resources sent to network according to their degree of relationship. SUR results.

	Sent to close family		Sent to extended family	Sent to extended family	Sent to neighbor	Sent to neighbor
	Controls	Controls and claims	Controls	Controls and claims	Controls	Controls and claims
Baseline: Standard dictator game (no claims, no hiding)						
	(1)	(2)	(3)	(4)	(5)	(6)
Claims	338.6*	503.1*	141.1	237.2	135.7	237.2
	(183.0)	(301.7)	(102.1)	(185.5)	(86.74)	(185.5)
Hiding and claims	-419.9	.	-149.4	.	-16.96	.
	(285.0)	.	(159.0)	.	(85.64)	.
Hiding and no claims	-319.0*	.	-225.7**	.	-70.79	.
	(169.7)	.	(94.67)	.	(81.10)	.
Age	-9.537	-7.602	5.073	1.891	-1.098	1.891
	(16.83)	(19.94)	(9.388)	(11.97)	(7.177)	(11.97)
Gender	-22.32	91.01	-52.18	-69.35	55.81	-69.35
	(129.1)	(175.4)	(72.01)	(107.6)	(61.94)	(107.6)
Religion	-23.55	-463.1*	0.296	-189.1	-5.539	-189.1
	(186.3)	(265.0)	(103.9)	(162.9)	(88.80)	(162.9)
Help parents	138.5	-30.87	94.05	112.1	63.72	112.1
	(130.4)	(204.8)	(72.71)	(125.3)	(62.57)	(125.3)
Land	0.287**	-2.927	0.327***	2.124	0.174***	2.124
	(0.129)	(6.728)	(0.0722)	(4.140)	(0.0621)	(4.140)
Risk aversion	37.13	22.88	18.16	-10.54	2.691	-10.54
	(43.79)	(69.14)	(24.43)	(42.43)	(20.98)	(42.43)
Experimental endowment	0.0675***	0.0453**	0.0274***	0.0142	0.0300***	0.0142
	(0.0140)	(0.0230)	(0.00783)	(0.0123)	(0.00672)	(0.0123)
Married	299.0	154.9	-14.16	-28.97		-28.97
	(254.7)	(333.0)	(142.1)	(204.8)		(204.8)
Claims received		0.219***		0.189***		0.205***
		(0.0423)		(0.0355)		(0.0528)
*N*	120	60	120	60	120	60

Standard errors in parentheses* p < 0.10, ** p < 0.05, *** p < 0.01. All specifications include year of study fixed effects. Constants not reported.

**Fig 2:**
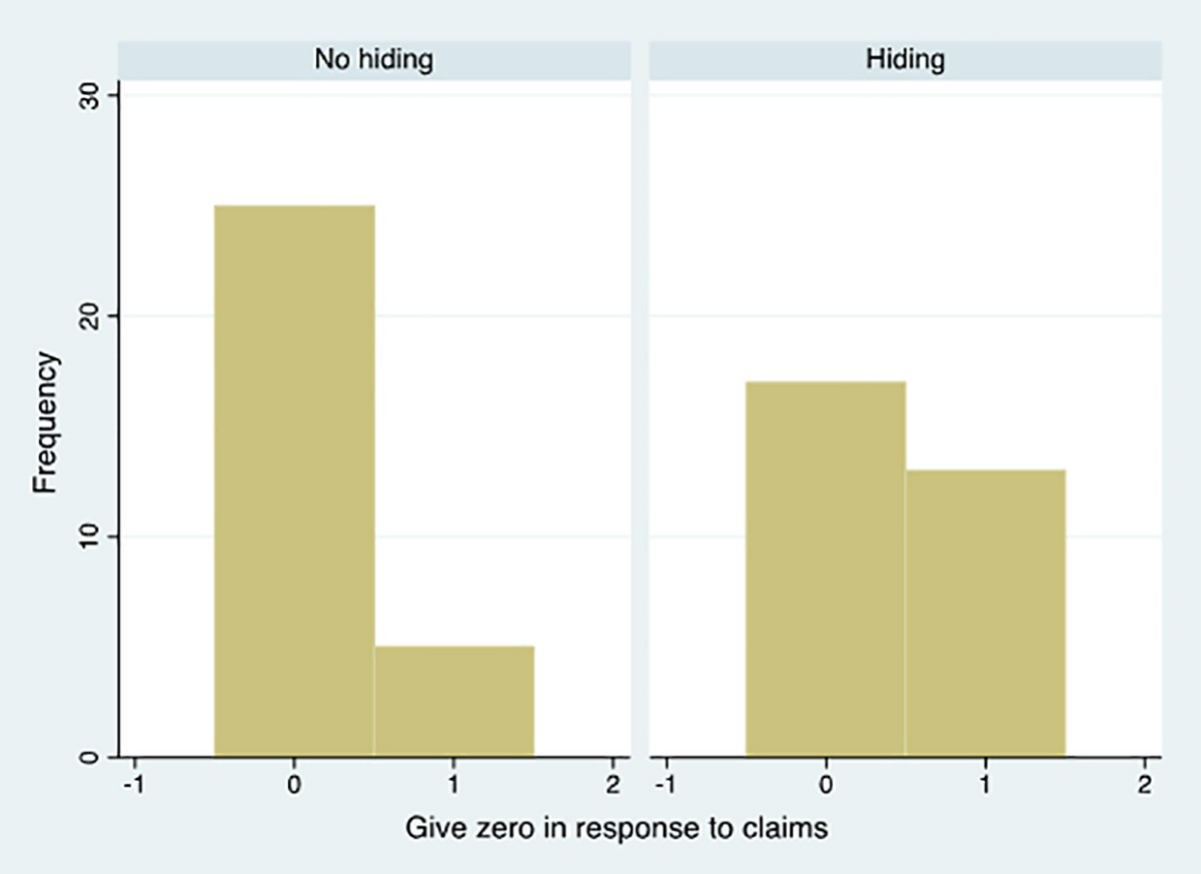
Frequency of zero resource given in response to claims. The impact of hiding.

Hiding does not only allow giving less in response to claims from the social network. It does also allow giving zero. Fig 2 reports the frequency of zero resources transferred in response to claims under the two different conditions *no hiding* and *hiding*.

Only 16% of participants give zero to members of the network when hiding is not possible. The remaining 84%, instead, shares resources. The difference is statistically significant at 5%. The difference is much less striking (and not statistically significant) when hiding is possible. In this condition, 43% of participants give zero while the remaining 57% share resources in response to claims.

### Investment decisions

We now turn to the investment analysis to investigate some of the possible economic implications of sharing redistributive pressure. We test how the share of the endowment that is invested is affected by different experimental conditions. *Hiding and not claim* is the reference. Table 6 column (1) and (2) shows the results of the OLS and Tobit regression respectively. The key idea is to test how exposure to sharing pressure may push individuals into lower investment decisions. We consider the condition no *claim* and *hiding* as the one where senders are exposed to the lowest level of pressure. They do not receive claims and they can also show to the others lower levels of income. This is the omitted category in the regression models. As above, we find that redistributive pressure does matter. All the conditions that entail being exposed to claims indeed are negatively correlated with both total investment and the share of resources that is invested. We also find that hiding provides the behavioral shelter to reduce sharing pressure.

**Table 6:** Investment analysis.

	Total investment—OLS	Share of the experimental endowment invested—Tobit
Baseline: no claims hiding		
	(1)	(2)
No hiding no claim	-588.1***	-0.0849*
	(51.44)	(0.0471)
Hiding and claim	-278.4***	-0.0403
	(71.52)	(0.0749)
No hiding and claim	-249.7***	-0.0451
	(65.22)	(0.0492)
Age	13.62	0.00226
	(8.697)	(0.00467)
Gender	376.9*	0.0832**
	(205.5)	(0.0358)
Religion	471.1***	0.0671
	(133.5)	(0.0517)
Help parents	-335.9	-0.0687*
	(221.3)	(0.0362)
Land	0.311***	0.0000567
	(0.0334)	(0.0000359)
Risk aversion	6.875	0.00818
	(42.64)	(0.0122)
Experimental endowment	0.0790***	0.0000145***
	(0.0272)	(0.00000390)
Married	82.63	0.00245
	(154.3)	(0.0707)
*N*	120	120
adj. *R*^2^	0.222	

Standard errors in parentheses* p < 0.10, ** p < 0.05, *** p < 0.01. Small cluster correction applied ([60]) in (1). All specifications include year of study fixed effects. Constant not reported. Session clustered standard errors in parentheses* p < 0.10, ** p < 0.05, *** p < 0.01. Small cluster correction applied ([60]). All specifications include year of study fixed effects. Constant not reported.

## 4. What is the social norm?

In general, there are three different sources of altruism in a context like the present one. First, altruism could be the manifestation of the Nash equilibrium of what is actually a repeated game, possibly unobserved by the researcher. People, thus, share some of their resources in one interaction because they expect to meet again. Second, it can be an intrinsic specification of subjects’ utility function: people feel better when sharing, because they are *intrinsically motivated* and have inbuilt in their utility function also the wellbeing of other subjects (see [61]). Third, it can be the result of a social norm that people follow. [62] is among the first to distinguish the causes of observed altruism in the context of the dictator game. Alger and Weibull [28, 46] discuss this at length in motivating their model of coerced altruism. More recently, [63] proposes a model based on pure altruism and shows how giving is affected by income. [64] distinguish between giving (moved by pure altruism) and giving in (moved by compliance to social norms). [65] highlighted the importance of social norms of redistribution, in Sub-Saharan Africa. [66] provided complementary evidence showing that in Tanzanian villages, individuals found it very difficult not to help one another. The experimental approach allows us to provide a possible explanation for the underlying psychological mechanism at play. Our experimental design rules out the first case: identities are anonymous and subjects cannot reciprocate. So, we can focus exclusively on the other two sources: *real* altruism or social norm of sharing. If subjects follow a social norm, it is also important to find evidence on how this rule of thumb actually works in the cognitive process of subjects.

### Sensitivity of type A subjects to the outcomes of the lottery

**Fig 3:**
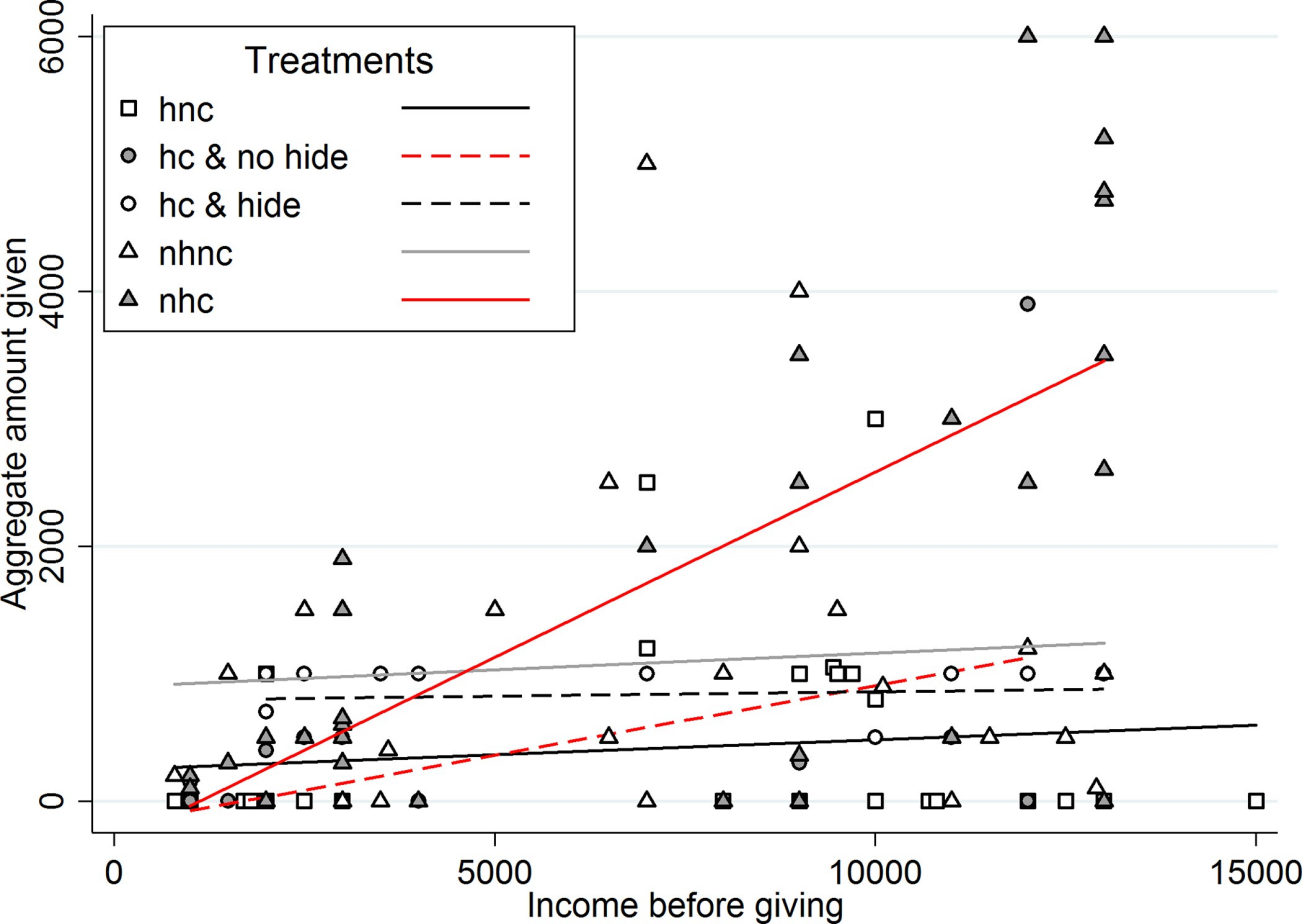
Income before giving vs aggregate amount given of all A types. Colors and linear fits are for the different treatments: hnc is “hide, no claim” (in this case all 30 A subjects decided to hide), nhnc is “no hide, no claim”, nhc is “no hide, claim”, and finally hc is “hide, claim”. In the fourth case 14 A subjects decided to hide, while the other 16 decided not to.

To obtain a first indication of the behavior of people, let us consider Fig 3 below. It does present a summary of the behavior of type A subjects. On the x-axis we have the aggregate income after the investment decision is made and outcome seen (minus the cost of hiding, if that choice was possible and taken), on the y-axis we have the aggregate amount of giving to the three type B subjects that the type A subject choses, possibly after claims have been made. First of all, note that while in the *hide* treatment, all type A subjects decided to hide, in the *hide and claim* treatment this choice was split. Overall, the linear fit shows that only those type A subjects that are facing simultaneously the *no hide* and the *claim* conditions seem to condition the amount given to type B subjects on the outcome of the investment. All the others seem indifferent. We ran regression with the same battery of controls used previously. For the 30 A subjects in the ‘nhc’ case the coefficient of income on quantity given is 0.25, positive at the 99% confidence level. For all the 44 subjects that are either in the ‘hiding and claiming and no hiding’ case or in the ‘no hiding and claiming’ case, the coefficient is 0.26, positive above the 99.9% confidence level. As is evident from Fig 3, in all the other cases (72 A subjects) we have that this coefficient is only 0.02 and is not statistically different from 0. This outcome seems to suggest that subjects, who are not expecting a claim (or expect a claim from people who do not know the outcome of the lottery), decide how much to give even before knowing the outcome of the investment and try to adhere to that choice.

The concept of ‘fairness’ that they adopt is social, and is independent of the (privately known only in case of success) outcome of the investment.

For this reason, we propose an approach based on a simple hypothesis, discussed here below.

### The *rational inattention* hypothesis

We focus on type A subjects—the decision makers. First of all we assume that a type A subject has the possibility to compute, on the basis of her endowment *w*, the amount $\tilde{y}(w)\leq w$, which is what she perceives as socially fair to give out, and depends on her own preferences, her perception of the social norms and on what she guesses that the other may think. For simplicity, we assume that $\tilde{y}(w)$ is linear in *w* (say, it is always 30% of *w*). Then, we assume that she has a utility function of the following form:

$$u(w,y)=w-y-\beta |y-\tilde{y}(w)|,$$

where *y* is the actual aggregate amount that she gives to B subjects, *β*>1 is a constant, and |∙| is just the absolute value. This functional form is a version of the benchmark on other regarding preferences from [67].

To be able to interpret the behavior of our subjects, we borrow from the literature on dual reasoning in decision making (see [68] for a recent survey) and suppose that the type A subject establishes what is the optimal *y* to give out before she actually knows the outcome of *w*: we call *y*_0_ the outcome of this first estimation. Since *β*>1 and $\tilde{y}(w)$ is linear, the optimal *y*_0_ computed at this stage will actually be equal to $\tilde{y}(E(w))$. Then, after the outcome *w* is realized, the decision maker may stick to the initial value $y_{0}=\tilde{y}(E(w))$, or re-compute $\tilde{y}(w)$, with the updated realized value of *w*. However, re-computing $\tilde{y}(w)$ comes at a (cognitive) cost. In particular, before starting to think about the problem, a decision maker decides non-rationally (we could say: *by instinct*) if she wants to use a low or high cognitive effort.

A very recent literature ([69–70] has formalized the concept of *rational inattention*. In this theoretical framework the decision maker tends to be biased towards ex-ante optimal actions, and if the information that she receives ex-post is not surprising, she will stick to the original decision (the choice of sticking to ex-ante optimal actions is called frictional decision making). In the literature, this typically happens if the stakes at play change (e.g., [71]) or if the framing of the problem changes (e.g., [72]), and the use of dual reasoning in intertemporal choices has been used by Fudenberg and Levine [73–74] to explain behavioral anomalies in consumption choices. In all these applications, the decision maker is supposed to be in isolation. Here, we want to understand under which circumstances the type A subjects will incur the cognitive cost of processing new information, or instead they will choose rational inattention and keep the choice that they made before the outcome of the lottery was known. We argue that in the context of our experiment, type A subjects, as decision makers, incur this cost only if there is a social pressure (a request from type B players) that is justified on hard evidence (i.e., the type B players know the outcome *w*), otherwise the decision maker will stick to *y*_0_.

It is important to stress one aspect. In some cases, not taking into account the new realized *w* may seem as an ex-post justification from the type A subjects: if they win in the lottery they have a psychological justification to give out less than they would do if they were re-processing the correct fair amount of $\tilde{y}(w)$. However, it is important to focus on those A subjects who actually loose in the lottery. From the design of our experiment, not re-computing $\tilde{y}(w)$ after a loss comes at a monetary cost, and this can be only justified, in the context of this modelling approach, assuming the cognitive cost that this new computation implies.

It is remarkable, from our experiment, that it is in the treatments *no hide and claim* and *hide and claim* (in the latter case, for those 16 subjects who decided not to hide) that not re-computing $\tilde{y}(w)$ after a loss comes at a monetary cost. It seems to us that this can be only justified, at least in the context of our modelling approach, assuming that this new computation implies a large cognitive cost.

### Consistency of the *rational inattention* hypothesis with the data

To test the implications of our hypothesis, we proceed in two steps, considering the shares of wealth given out by the type A subjects under different treatments, choices, and outcomes of the lottery.

First, we compare the cases where the combination of no hiding and claim is not present at a cost for the subjects (i.e., all the subjects in the treatment *no hide and claim* and the 14 subjects who decided to hide in the treatment *hide and claim*) with those 30 type A subjects who cannot hide but receive no claim. In all these cases, the type A subjects turns out to be indifferent to the outcome of the lottery when giving. However, the first 44 have paid for hiding and they seem to externalize this cost to the type B subjects giving less to them. The amount given by the 44 subjects who pay for hiding was around 3% (2% to external, 2% to neighbors, 5% to close family members); instead the amount given by the 33 subjects who receive no claims for free was around 6% (6% to external, 3% to neighbors, 8% to close family members). Applying Kruskal-Wallis equality-of-populations rank test, thes average outcomes are statistically different at 90%, even if they are not independently in the 3 separated cases.

This outcome seems not consistent with pure altruistic motivations (because paying for hiding is a waste for both players), but is consistent with the fact that some type A subjects try to avoid the pressure for giving out because of social norms, even if it comes at a cost for the type B subjects. Then, to better understand what drives or dual reasoning approach, we study those type A subjects who actually lost the lottery. If we consider the treatment *hide and claim*, where 16 subjects chose not to hide (among them 11 lost the investment) while 14 chose to hide (among them 7 lost the investment). The amount given by the 11 unlucky subjects who decided not to hide was around 2% (2% to external, 1% to neighbors, 3% to close family members), instead the amount given by the 7 unlucky subjects who decided to hide was around 10% (10% to external, 7% to neighbors, 13% to close). Even with these low numbers, applying Kruskal-Wallis equality-of-populations rank test, the average outcomes are statistically different at 99%, even independently in the 3 separated cases of external, neighbors and close. In principle, choice could depend also on the amount that is lost. However, our observations are too few to make any differentiated claim for those subjects who lost more or less of their resources in the lottery. This indicates that those subjects who decided to hide stick to a ‘fair’ proportion of their wealth to share with the others, independently on the outcome of the lottery. This result is consistent with a dual reasoning approach, as the one we propose above, where the A subject switch from a *low* to a *high* cognitive effort, after the outcome of the lottery, only if they know that the B subjects are aware of this outcome.

Moreover, and still on this second point, comparing the two treatments *nhc* and *nhnc*, We see that those who lost in the *nhc* treatment provide significantly less amount to the type B subjects than those who lost in the *nhnc* treatment: in both cases they know that the type B subjects know that they lost the lottery, but in the latter case the type B subjects are not going to ask for a specific amount. We compare 7 A subjects who lost the lottery in the nhnc case with the 13 A subjects who lost the lottery in the nhnc case. Even with these low numbers, applying Kruskal-Wallis equality-of-populations rank test, the average share of wealth given out to B subjects are statistically different at 90%, independently in the 3 separated cases of external, neighbors and close. So, according to the dual reasoning approach, what makes the type A subjects switch to a *high* cognitive approach and re-compute what would be fair to give out, is an actual request from the type B subjects, which is based on hard evidence. This re-computation is made even when it comes at a monetary cost for them, and more importantly, even if that would not be perceived as unfair by the type B subjects.

Overall, this shows how strong the social norms can be, as they may induce the economic agents to stick their actions to predetermined choices, even if this can be socially inefficient or even detrimental for them.

Summing up, there is supporting evidence for the conjecture that type A subjects decide ex-ante to base their giving on the outcome of the investment only if they face claims that are based on hard evidence of this outcome. Otherwise, they decide before the outcome of the investment is known how much to give. If they have spent money to hide, and so to avoid the *no hide and claim* situation, they externalize this cost within the amount they give. This conjecture seems to describe a behavior that is based on social norms, and seems to support the possibility that subjects are not only driven by intrinsic motivation, in the form of utility derived from the actual allocation of resources among themselves and their peers.

## 5. Conclusions

Social networks in the developing world, such as kinship or extended families, are characterized by high level of pro sociality of their members. Individuals share resources and obtain a wide range of important services from the network, such as insurance and credit, labor inputs, when markets are imperfect or absent. An important feature of these networks is that they are characterized by the moral imperative to share resources among their members. In this paper we presented the results of an experiment conducted in rural Tanzania. The experiment is framed as a typical social network in a developing country with close links to kinship and extended family. We designed a novel modified dictator game with multiple recipients where in some treatments the subjects are allowed to claim some of the resources from the senders they are related to. The senders in some treatments had, instead, the possibility to hide their resources at a cost. This allows the testing behavioral responses both in terms of investment and giving decision under redistributive pressure. We find experimental evidence highlighting the detrimental impact of sharing pressure on the likelihood of undertaking investments. These are, indeed, smaller when the possibility of redistributive pressure is larger. We also documented how important the sharing norms are in dictating giving and how individuals may engage in costly activities such as hiding to prevent resource reallocation. We establish a link between hiding and pressure avoidance in the context of risky investments, and we explain its rationale with the help of a hypothesis based on rational inattention.

From a societal perspective, the redistributive pressure may result in a significant negative impact on economic development; either directly through less investment and costly hiding activities or indirectly since fewer resources are available for investment due to sharing or both ([2, 18–19, 36, 75]). Hiding is a behavioral shelter. It provides the possibility to reduce the amount of giving and reduce social network pressure to redistribute resources. In our context, hiding provides a way to shelter against giving in response to redistributive pressure supporting that giving within the network seems in fact driven by sense of duty and sharing norms. Our results contribute to this debate by showing the underlying behavioral mechanisms, their consequences in terms of sharing and in terms of investment. Findings are consistent with dual reasoning approaches.

At this stage some caveats are necessary. First, like in any study of this nature, further experimental research is needed to scale up and generalize our results in variety of different settings (e.g., different countries, lab in the field etc.). Second, it would be extremely important to study how different degrees of uncertainty or the size of the network may quantitatively or qualitatively the results. Future research should address these issues as well as studying more in depth how improving market access to credit or insurance may affect the patterns that we have documented in this paper.

## Supporting information

S1 Table Summary statistics of control variables. (DOCX)

S2 Table Balance test. (DOCX)

S1 File Instructions. (DOCX)

S1 Dataset (DTA)
