# Supplementary material for: Altruism and the pressure to share: Lab evidence from Tanzania
Source: PLoS One. 2019 May 21;14(5):e0212747. doi: 10.1371/journal.pone.0212747 (PMC6529004; doi:10.1371/journal.pone.0212747)
Supplement: S1 Table — (DOCX) [file pone.0212747.s001.docx]

**S1 Table.** **Summary statistics of control variables.**

| Variable | Definition | Mean | Std. Dev. | Min | Max |
| --- | --- | --- | --- | --- | --- |
| Age | Age of the participant in the experiment | 25.31 | 5.01 | 19 | 49 |
| Male | Gender of the participant in the experiment; 1=male; 0=otherwise | 0.72 | 0.44 | 0 | 1 |
| Religion | Religion of the participant in the experiment; 1=Christian; 0=otherwise | 0.89 | 0.308 | 0 | 1 |
| Help parents | Answer to the following question: do you help your parents in their business activities?  1=yes; 0=otherwise | 0.707 | 0.455 | 0 | 1 |
| Land | Answer to the following question: how much land your family owns in hectares? | 6.53 | 10.71 | 0 | 80 |
| Married | Status of the participant; 1=Yes; 0=otherwise | 0.46 | 0.49 | 0 | 1 |
| Experimental endowment | Endowment determined in investment game play in the experiment | 6923 | 4187 | 800 | 1500 |
| Risk Aversion | Risk aversion of the participant. Scale from 1 (risk averse) to 6 (extremely risk lover). | 3.71 | 1.37 | 1 | 6 |
