# Supplementary material for: Altruism and the pressure to share: Lab evidence from Tanzania
Source: PLoS One. 2019 May 21;14(5):e0212747. doi: 10.1371/journal.pone.0212747 (PMC6529004; doi:10.1371/journal.pone.0212747)
Supplement: S2 Table — (DOCX) [file pone.0212747.s002.docx]

**S2 Table.** Balance test

|  | Hiding | No Hiding | Pr(\|T\| > \|t\|) | Claims | No Claims | Pr(\|T\| > \|t\|) |
| --- | --- | --- | --- | --- | --- | --- |
| Age | 24.7 | 25.46 | 0.307 | 27.48 | 23.05 | 0.00 |
| Gender | 0.6 | 0.76 | 0.01 | 0.71 | 0.73 | 0.77 |
| Religion | 0.9 | 0.8 | 0.54 | 0.89 | 0.9 | 0.83 |
| Risk aversion | 3.55 | 3.74 | 0.35 | 3.8 | 3.5 | 0.2 |
| Land | 8.38 | 8.95 | 0.12 | 7.19 | 5.87 | 0.35 |
| Help parents | 0.68 | 0.72 | 0.58 | 0.79 | 0.62 | .003 |
| Married | 0.53 | 0.39 | 0.056 | 0.81 | 0.041 | 0.00 |
