# Supplementary material for: Altruism and the pressure to share: Lab evidence from Tanzania
Source: PLoS One. 2019 May 21;14(5):e0212747. doi: 10.1371/journal.pone.0212747 (PMC6529004; doi:10.1371/journal.pone.0212747)
Supplement: S1 File — (DOCX) [file pone.0212747.s003.docx]

**Altruism and the Pressure to Share: Lab Evidence from Tanzania**

**Supporting Material – experimental instructions**

**Instructions A players**

**Room 1**

Thank you for taking part in this economic game. It is important that you take your time to understand the instructions. The instructions are distributed to you are for your private information. **Please do not communicate with the other participants during the game. This is like an exam!** Should you have any questions please raise your hand. The remainder of these instructions will explain exactly how this game is run. Since in the game you will have to make a private decision, we ask that you not tell anyone your decision either during, or after, the game. To keep anonymity you have been assigned a game ID. For instance A1, A2, A3 etc. if you are sitting in room A or B1, B2, B3 etc. if you are sitting in room B. You will not be told who these people are neither during nor after the game.

This game is played in groups. You have been linked with 3 other persons and they are sitting in a different room from yours. Imagine that these three persons are related to you. One is a member of your close family (your brother or your sister for example), one is a member of your extended family (your cousin, for example), and last one is your neighbor. You may think of it as your social network.

In the game you will have make some decisions. Your decisions have economic implications in the sense that you can make some earnings or not. You will take home with you the money you have at the end of the game. During the experiment we shall not speak of Shilling, but of Experimental Currency Units (ECU). Your entire earnings will be calculated in ECU. At the end of the experiment the total amount of ECUs you have earned will be converted to Schillings at the rate of **1000 ECU = 1000 Shilling** and will be immediately paid to you in cash. The different stages of the game are summarized as follows.

**1. Private Decisions in Room 1**

You will be given some money (5000 ECU) that you will divide between a saving box and an investment box. The money is yours. How to divide the money between the two boxes is your first decision in the game. The money that you put in the investment box can be used to generate more money. The money can also be lost. So if the investment is successful you will get back more money that you did put in the investment box. If the investment fails you loose all the money you put it in the investment box. The money that you put in the saving box just sits there. It does not generate more money. You do not loose either.

How do we determine the result of the investment? After that you divide your 5000 ECU between the saving box and the investment box, we will ask you to shake a coin. If the coin lands with **head facing up** you will be paid three times the amount of money that you put in the investment box. If the coin lands with **tail facing up** you loose the amount you put in the investment box.

So, for example, if you decide to put 3000 ECU in the investment box how much will you get? You will toss the coin and determine it. If the coin lands with head facing up you will get 3x3000 ECU = 9000 ECU. If the coin land with tail facing up you get 0. You can put as much or as little you want in the investment cup. If you like you can put everything (all the 5000 ECU) or nothing (0 ECU) in the investment box. If you like you can put everything (all the 5000 ECU) or nothing (0 ECU) in the saving box. Your close family, extended family and neighbor will know your income. So they will know how much you have in your saving box and how much you obtained from your investment - if you have put any money in the investment box. If you made a loss they will know that you have 0 in the investment box.

First example, if you decided to put in the investment box 1000 ECU and gained and 4000 ECU in the saving box. Your income is 6000 ECU. Your close family, extended family and neighbors will be informed about it. Second example, you decided to put 4000 ECU in the investment box and gained. Your total income is 13000 ECU. Again your social network will be informed. Here is a sketch of the game:

TOTAL ECU = ECU the saving box + outcome from investment box

Total ECU will be then disseminated within the network

**Write down in the decision sheet provided how much you have in the two different boxes.** A member of the team will be with you and let you toss the coin to determine the amount in the investment box and saving box. Write down the two different amounts.

**2. Dissemination stage**

A member of the team will collect the information about your money. The information about your money will be disseminated in your social network.

Example 1: you divided your initial 5000 ECU into 1000 ECU in the investment box and 4000 ECU in the saving box. You toss the coin and obtain tail. So you lost 1000 ECU. Your neighbor, extended family and close family will be told that 4000 ECU is your final income.

Example 2: you divided your initial 5000 ECU into 3000 in the investment box and 2000 ECU. You toss the coin and obtain head. So you gained 3x3000 and your final amount is 11000 ECU. Your close family, extended family, neighbors are informed that you have 11000 ECU.

Now we ask you to fill the questionnaire that we provided to you. Once you have finished please put it on the side.

**3. Giving stage**

The members of your group (your close family, your extended family, your neighbor) have now full information on your income. They sit in a different room. Their income is 2000 ECU each. So your close family member has 2000 ECU, your extended family has 2000 ECU, your neighbor has 2000 ECU. If you wish you can give them money. The amount of income you may give depends upon the relationship you have. Close family (your brother or your sister for instance) you may give up to 50% of your money. Your extended family may give up to 30%. Your neighbor may give up to 20% of your money.

**4. Final pay off stage**

At this stage of the game you know how much money you have in the saving box and the investment box. You know, how much your close family, your extended family, and your neighbor have (they all have 2000 ECU). Your final decision is how much to give to them. You can give all of them, to some of them or to none. You can give as much or as little as you want (zero ECU included). This is your final private decision. How much is left after this decision is for you to take home.

**Thank you for playing – you can leave the room.**

**Payments will be done in private.** Questions?

**Summary of the decisions to be taken during the game**

**1 Decision at the beginning.** How much of the 5000 ECU you want to allocate between saving box and investment box.

**1 Decision at the pay off stage.** How much to pay in response of the claims of the close family, the extended family, the neighbours. Can be as much or as little you want (zero included).

**Do you have any questions? Please raise your hand; a member of the research team will come to you and answer your question in private.**

**Room 2**

Thank you for taking part in this economic game. It is important that you take your time to understand the instructions. The instructions which are distributed to you are for your private information. **Please do not communicate with the other participants during the game. This is like an exam!** Should you have any questions please raise your hand. The remainder of these instructions will explain exactly how this game is run. Since in the game you will have to make a private decision, we ask that you not tell anyone your decision either during, or after, the game. To keep anonymity you have been assigned a game ID. For instance C1, C2, C3 etc. if you are sitting in room C or D1, D2, D3 etc. if you are sitting in room D. You will not be told who these people are neither during nor after the game.

This game is played in groups. You have been linked with 3 other persons and they are sitting in a different room from yours. Imagine that these three persons are related to you. One is a member of your close family (your brother or your sister for example), one is a member of your extended family (your cousin, for example), and last one is your neighbor. You may think of it as your social network.

In the game you will have make some decisions. Your decisions have economic implications in the sense that you can make some earnings or not. You will take home with you the money you have at the end of the game. During the experiment we shall not speak of Shilling, but of Experimental Currency Units (ECU). Your entire earnings will be calculated in ECU. At the end of the experiment the total amount of ECUs you have earned will be converted to Schillings at the rate of **1000 ECU = 1000 Shilling** and will be immediately paid to you in cash. The different stages of the games are summarized as follows.

**1. Private Decisions in Room 2**

You will be given some money (5000 ECU) that you will divide between a saving box and an investment box. The money is yours. How to divide the money between the two boxes is your first decision. The money that you put in the investment box can be used to generate more money. The money can also be lost. So if the investment is successful you will get back more money that you did put in the investment box. If the investment fails you loose all the money you put it in the investment box. The money that you put in the saving box just sits there. It does not generate more money. You do not loose either.

How do we determine the result of the investment? After that you divide your 5000 ECU between the saving box and the investment box, we will ask you to shake a coin. If the coin lands with **head facing up** you will be paid three times the amount of money that you put in the investment box. If the coin lands with **tail facing up** you loose the amount you put in the investment box.

So, for example, if you decide to put 3000 ECU in the investment box how much will you get? You will toss the coin and determine it. If the coin lands with head facing up you will get 3x3000 ECU = 9000 ECU. If the coin land with tail facing up you get 0. You can put as much or as little you want in the investment box. If you like you can put everything (all the 5000 ECU) or nothing (0 ECU) in the investment box. If you like you can put everything (all the 5000 ECU) or nothing (0 ECU) in the saving box. Your close family, extended family and neighbor will know your income. So they will know how much you have in your saving box and how much you obtained from your investment - if you have put any money in the investment box. If you made a loss they will know that you have 0 in the investment box.

You are given the opportunity, however, to pay a fee and avoid to inform your network about your true total amount of money. They will be told that you have 5000 ECU in the saving box and nothing more. We will not reveal your decision to the rest of your network. You will not have to share any information on the amount of money you may have invested. You must decide this before that the result of the investment is known to you. First example, if you decided to put in the investment box 2000 ECU and gained and 3000 ECU in the saving box. Your income is 3x2000 ECU+ 3000 ECU. In total you will have 9000 ECU. You paid a fee and informed your network that your income is instead 5000 ECU. Second example, you decided to put 4000 ECU in the investment box and gained. Your total income is 13000 ECU. You paid a fee and informed the close family, the extended family and the neighbor that you have only 5000 ECU in the saving box. Third example, you have divided your 5000 ECU in the following way. 2000 ECU in the investment box and 3000 ECU in the saving box. You toss the coin and lost (tail facing up). Your final income is 3000 ECU. You decide not to pay any fee and inform your network that you have 3000 ECU.

To recap: you have to choose how to allocate your 5000 ECU between the saving box and the investment box and if you want to avoid to inform the others.

How much is the fee? The fee is 500 if you want to hide from your close family your income, 300 ECU if you want to hide from your extended family your income, 200 ECU if you want to conceal your amount from your neighbor. You can decide not to pay the fees and disclose the full amount in the saving box and in the investment box. You can decide to pay the fees only for some members of the network and not other. For instance, if you do not want to disclose your full amount to your cousins and neighbor you will pay 500 ECU. Or if you do not want to disclose your full amount to your neighbor, but are happy with full disclosure to your family (close and extended) you pay a fee of 200 ECU. It is entirely up to you. It is your decision in the game. Got it? If not clear raise your hand now. Here is it a sketch of this stage of the game.

TOTAL ECU = ECU the saving box + outcome from investment box

Total ECU will be disseminated within the network if fee not paid. Otherwise some or all in the network will be told that you have only 5000 ECU.

**Write down in the decision sheet provided how much you have in the two different boxes.** A member of the team will be with you and let you toss the coin to determine the amount in the investment box and saving box. Write down the two different amounts.

**Write down in the decision sheet provided if you wish to pay fee to avoid full disclosure of the amount of money you have placed in the saving box and in the investment box.** If you wish to pay a fee, decide to whom you want to avoid to tell the full amount. Your close family, your extended family, your neighbor. Some of them, all of them you can freely choose.

**2. Dissemination stage**

A member of the team will collect the information about your income (full disclosure or not) and will disseminated in your social network.

Example 1: you divided your initial 5000 ECU into 1000 ECU in the investment box and 4000 ECU in the saving box. You toss the coin and obtain tail. So you lost 1000 ECU. You did not want to pay any fee and wanted everybody to know your income (full disclosure). Your neighbor, extended family and close family will be told that 4000 ECU is your final income.

Example 2: you divided your initial 5000 ECU into 3000 in the investment box and 2000 ECU. You toss the coin and obtain head. So you gained 3x3000 and your final amount is 11000 ECU. You wanted your full amount not to be disclosed to your extended family. You paid 300 ECU fee. Your money is 10700 ECU.

**Now we ask you to fill the questionnaire that we provided to you. Once you have finished please put it on the side.**

**3. Give stage**

The members of your group (your close family, your extended family, your neighbor) have now information on your income (full or partial depending on your decision). They sit in a different room. Their income is 2000 ECU each. So your close family member has 2000 ECU, your extended family has 2000 ECU, your neighbor has 2000 ECU. If they wish you can give them money. The amount of income you may give depends upon the relationship you have. Close family (your brother or your sister for instance) you may give up to 50% of your money. Your extended family may give up to 30%. Your neighbor may give up to 20% of your money.

**4. Final pay off stage**

At this stage of the game you know how much money you have in the saving box and the investment box. You know, how much your close family, your extended family, and your neighbor have (they all have 2000 ECU). Your final decision is how much to give to them. You can give to all of them, to some of them or to none. You can give as much or as little as you want. This is your final private decision. How much is left after this decision is for you to take home.

**Thank you for playing – you can leave the room.**

**Payments will be done in private.** Questions?

**Summary of the decisions to be taken during the game**

**2 decisions at the beginning.** How much of the 5000 ECU you want to allocate between saving box and investment box. Pay a fee to avoid that the amount of money you hold is fully disclosed to your close family, your extended family, your neighbour. Some of them or none of them.

**1 Decision at the pay off stage.** How much to pay to the close family, the extended family, the neighbours. Can be as much or as little you want (zero included).

**Do you have any questions? Please raise your hand; a member of the research team will come to you and answer your question in private.**

**Room 3**

Thank you for taking part in this economic game. It is important that you take your time to understand the instructions. The instructions are distributed to you are for your private information. **Please do not communicate with the other participants during the game. This is like an exam!** Should you have any questions please raise your hand. The remainder of these instructions will explain exactly how this game is run. Since in the game you will have to make a private decision, we ask that you not tell anyone your decision either during, or after, the game. To keep anonymity you have been assigned a game ID. For instance A1, A2, A3 etc. if you are sitting in room A or B1, B2, B3 etc. if you are sitting in room B. You will not be told who these people are neither during nor after the game.

This game is played in groups. You have been linked with 3 other persons and they are sitting in a different room from yours. Imagine that these three persons are related to you. One is a member of your close family (your brother or your sister for example), one is a member of your extended family (your cousin, for example), and last one is your neighbor. You may think of it as your social network.

In the game you will have make some decisions. Your decisions have economic implications in the sense that you can make some earnings or not. You will take home with you the money you have at the end of the game. During the experiment we shall not speak of Shilling, but of Experimental Currency Units (ECU). Your entire earnings will be calculated in ECU. At the end of the experiment the total amount of ECUs you have earned will be converted to Schillings at the rate of **1000 ECU = 1000 Shilling** and will be immediately paid to you in cash. The different stages of the games are summarized as follows.

**1. Private Decisions in Rooms 3**

You will be given some money (5000 ECU) that you will divide between a saving box and an investment box. The money is yours. How to divide the money between the two boxes is your first decision in the game. The money that you put in the investment box can be used to generate more money. The money can also be lost. So if the investment is successful you will get back more money that you did put in the investment box. If the investment fails you loose all the money you put it in the investment box. The money that you put in the saving box just sits there. It does not generate more money. You do not loose either.

How do we determine the result of the investment? After that you divide your 5000 ECU between the saving box and the investment box, we will ask you to shake a coin. If the coin lands with **head facing up** you will be paid three times the amount of money that you put in the investment box. If the coin lands with **tail facing up** you loose the amount you put in the investment box.

So, for example, if you decide to put 3000 ECU in the investment box how much will you get? You will toss the coin and determine it. If the coin lands with head facing up you will get 3x3000 ECU = 9000 ECU. If the coin land with tail facing up you get 0. You can put as much or as little you want in the investment cup. If you like you can put everything (all the 5000 ECU) or nothing (0 ECU) in the investment box. If you like you can put everything (all the 5000 ECU) or nothing (0 ECU) in the saving box. Your close family, extended family and neighbor will know your income. So they will know how much you have in your saving box and how much you obtained from your investment - if you have put any money in the investment box. If you made a loss they will know that you have 0 in the investment box.

First example, if you decided to put in the investment box 1000 ECU and gained and 4000 ECU in the saving box. Your income is 6000 ECU. Your close family, extended family and neighbors will be informed about it. Second example, you decided to put 4000 ECU in the investment box and gained. Your total income is 13000 ECU. Again your social network will be informed. Here is a sketch of the game:

TOTAL ECU = ECU the saving box + outcome from investment box

Total ECU will be then disseminated within the network

**Write down in the decision sheet provided how much you have in the two different boxes.** A member of the team will be with you and let you toss the coin to determine the amount in the investment box and saving box. Write down the two different amounts.

**2. Dissemination stage**

A member of the team will collect the information about your money and will disseminated in your social network.

Example 1: you divided your initial 5000 ECU into 1000 ECU in the investment box and 4000 ECU in the saving box. You toss the coin and obtain tail. So you lost 1000 ECU. Your neighbor, extended family and close family will be told that 4000 ECU is your final income.

Example 2: you divided your initial 5000 ECU into 3000 in the investment box and 2000 ECU. You toss the coin and obtain head. So you gained 3x3000 and your final amount is 11000 ECU. Your close family, extended family, neighbors are informed that you have 11000 ECU.

Now we ask you to fill the questionnaire that we provided to you. Once you have finished please put it on the side.

**3. Claims stage**

The members of your group (your close family, your extended family, your neighbor) have now full information on your income. They sit in a different room. Their income is 2000 ECU each. So your close family member has 2000 ECU, your extended family has 2000 ECU, your neighbor has 2000 ECU. If they wish they can make a claim to you. They may ask some of your money. The amount of income they may ask depends upon the relationship you have. Close family (your brother or your sister for instance) may ask up to 50% of your money. Your extended family may ask up to 30%. Your neighbor may ask up to 20% of your money.

The game team will bring this information back to you. They will inform you how much your social group wants from you.

**Please write down in the decision sheet how much your close family, extended family and neighbor are asking from you.**

**4. Final pay off stage**

At this stage of the game you know how much money you have in the saving box and the investment box. You know, how much your close family, your extended family, and your neighbor have (they all have 2000 ECU). You also know how much they ask from you. Your final decision is how much to give back to them in response. You can give back to all of them, to some of them or to none. You can give as much or as little as you want (zero ECU included). This is your final private decision. How much is left after this decision is for you to take home.

**Thank you for playing – you can leave the room.**

**Payments will be done in private.** Questions?

**Summary of the decisions to be taken during the game**

**1 Decision at the beginning.** How much of the 5000 ECU you want to allocate between saving box and investment box.

**1 Decision at the pay off stage.** How much to pay in response of the claims of the close family, the extended family, the neighbours. Can be as much or as little you want (zero included).

**Do you have any questions? Please raise your hand; a member of the research team will come to you and answer your question**

**Room 4**

Thank you for taking part in this economic game. It is important that you take your time to understand the instructions. The instructions which are distributed to you are for your private information. **Please do not communicate with the other participants during the game. This is like an exam!** Should you have any questions please raise your hand. The remainder of these instructions will explain exactly how this game is run. Since in the game you will have to make a private decision, we ask that you not tell anyone your decision either during, or after, the game. To keep anonymity you have been assigned a game ID. For instance C1, C2, C3 etc. if you are sitting in room C or D1, D2, D3 etc. if you are sitting in room D. You will not be told who these people are neither during nor after the game.

This game is played in groups. You have been linked with 3 other persons and they are sitting in a different room from yours. Imagine that these three persons are related to you. One is a member of your close family (your brother or your sister for example), one is a member of your extended family (your cousin, for example), and last one is your neighbor. You may think of it as your social network.

In the game you will have make some decisions. Your decisions have economic implications in the sense that you can make some earnings or not. You will take home with you the money you have at the end of the game. During the experiment we shall not speak of Shilling, but of Experimental Currency Units (ECU). Your entire earnings will be calculated in ECU. At the end of the experiment the total amount of ECUs you have earned will be converted to Schillings at the rate of **1000 ECU = 1000 Shilling** and will be immediately paid to you in cash. The different stages of the games are summarized as follows.

**1. Private Decisions in Rooms 4**

You will be given some money (5000 ECU) that you will divide between a saving box and an investment box. The money is yours. How to divide the money between the two boxes is your first decision. The money that you put in the investment box can be used to generate more money. The money can also be lost. So if the investment is successful you will get back more money that you did put in the investment box. If the investment fails you loose all the money you put it in the investment box. The money that you put in the saving box just sits there. It does not generate more money. You do not loose either.

How do we determine the result of the investment? After that you divide your 5000 ECU between the saving box and the investment box, we will ask you to shake a coin. If the coin lands with **head facing up** you will be paid three times the amount of money that you put in the investment box. If the coin lands with **tail facing up** you loose the amount you put in the investment box.

So, for example, if you decide to put 3000 ECU in the investment box how much will you get? You will toss the coin and determine it. If the coin lands with head facing up you will get 3x3000 ECU = 9000 ECU. If the coin land with tail facing up you get 0. You can put as much or as little you want in the investment box. If you like you can put everything (all the 5000 ECU) or nothing (0 ECU) in the investment box. If you like you can put everything (all the 5000 ECU) or nothing (0 ECU) in the saving box. Your close family, extended family and neighbor will know your income. So they will know how much you have in your saving box and how much you obtained from your investment - if you have put any money in the investment box. If you made a loss they will know that you have 0 in the investment box.

You are given the opportunity, however, to pay a fee and avoid to inform your network about your true total amount of money. They will be told that you have 5000 ECU in the saving box and nothing more. We will not reveal your decision to the rest of your network. You will not have to share any information on the amount of money you may have invested. First example, if you decided to put in the investment box 2000 ECU and gained and 3000 ECU in the saving box. Your income is 3x2000 ECU+ 3000 ECU. In total you will have 9000 ECU. You paid a fee and informed your network that your income is instead 5000 ECU. Second example, you decided to put 4000 ECU in the investment box and gained. Your total income is 13000 ECU. You paid a fee and informed the close family, the extended family and the neighbor that you have only 5000 ECU in the saving box. Third example, you have divided your 5000 ECU in the following way. 2000 ECU in the investment box and 3000 ECU in the saving box. You toss the coin and lost (tail facing up). Your final income is 3000 ECU. You decide not to pay any fee and inform your network that you have 3000 ECU.

To recap: you have to choose how to allocate your 5000 ECU between the saving box and the investment box and if you want to avoid to inform the others.

How much is the fee? The fee is 500 if you want to hide from your close family your income, 300 ECU if you want to hide from your extended family your income, 200 ECU if you want to conceal your amount from your neighbor. You can decide not to pay the fees and disclose the full amount in the saving box and in the investment box. You can decide to pay the fees only for some members of the network and not other. For instance, if you do not want to disclose your full amount to your cousins and neighbor you will pay 500 ECU. Or if you do not want to disclose your full amount to your neighbor, but are happy with full disclosure to your family (close and extended) you pay a fee of 200 ECU. It is entirely up to you. It is your decision in the game. Got it? If not clear raise your hand now. Here is it a sketch of this stage of the game.

TOTAL ECU = ECU the saving box + outcome from investment box

Total ECU will be disseminated within the network if fee not paid. Otherwise some or all in the network will be told that you have only 5000 ECU.

**Write down in the decision sheet provided how much you have in the two different boxes.** A member of the team will be with you and let you toss the coin to determine the amount in the investment box and saving box. Write down the two different amounts.

**Write down in the decision sheet provided if you wish to pay fee to avoid full disclosure of the amount of money you have placed in the saving box and in the investment box.** If you wish to pay a fee, decide to whom you want to avoid to tell the full amount. Your close family, your extended family, your neighbor. Some of them, all of them you can freely choose.

**2. Dissemination stage**

A member of the team will collect the information about your income (full disclosure or not) and will disseminated in your social network.

Example 1: you divided your initial 5000 ECU into 1000 ECU in the investment box and 4000 ECU in the saving box. You toss the coin and obtain tail. So you lost 1000 ECU. You did not pay any fee and as wanted everybody to know your income (full disclosure). Your neighbor, extended family and close family will be told that 4000 ECU is your final income.

Example 2: you divided your initial 5000 ECU into 3000 in the investment box and 2000 ECU. You toss the coin and obtain head. So you gained 3x3000 and your final amount is 11000 ECU. You wanted your full amount not to be disclosed to your extended family. You paid 300 ECU fee. Your money is 10700 ECU.

**Now we ask you to fill the questionnaire that we provided to you. Once you have finished please put it on the side.**

**3. Claims stage**

The members of your group (your close family, your extended family, your neighbor) have now information on your income (full or partial depending on your decision). They sit in a different room. Their income is 2000 ECU each. So your close family member has 2000 ECU, your extended family has 2000 ECU, your neighbor has 2000 ECU. If they wish they can make a claim to you. They may ask some of your money. The amount of income they may ask depends upon the relationship you have. Close family (your brother or your sister for instance) may ask up to 50% of your money. Your extended family may ask up to 30%. Your neighbor may ask up to 20% of your money.

The game team will bring this information back to you. They will inform you how much your social group wants from you by writing the amount in the Decision sheet you are provided.

**4. Final pay off stage**

At this stage of the game you know how much money you have in the saving box and the investment box. You know, how much your close family, your extended family, and your neighbor have (they all have 2000 ECU). You also know how much they ask from you (based on your decision to fully disclose or not). Your final decision is how much to give back to them in response. You can give back to all of them, to some of them or to none. You can give as much or as little as you want. This is your final private decision. How much is left after this decision is for you to take home.

**Thank you for playing – you can leave the room. Payments will be done in private.** Questions?

**Summary of the decisions to be taken during the game**

**2 decisions at the beginning.** How much of the 5000 ECU you want to allocate between saving box and investment box. Pay a fee to avoid that the amount of money you hold is fully disclosed to your close family, your extended family, your neighbour. Some of them or none of them.

**1 Decision at the pay off stage.** How much to pay in response of the claims of the close family, the extended family, the neighbours. Can be as much or as little you want (zero included).

**Do you have any questions? Please raise your hand; a member of the research team will come to you and answer your question in private.**

**Instructions for B players**

**Room 5**

Thank you for taking part in this economic game. It is important that you take your time to understand the instructions. The instructions which are distributed to you are for your private information. **Please do not communicate with the other participants during the game. This is like an exam!** Should you have any questions please raise your hand. The remainder of these instructions will explain exactly how this game is run. Since in the game you will have to make a private decision, we ask that you not tell anyone your decision either during, or after, the game. To keep anonymity you have been assigned a game ID. For instance A1, A2, A3 etc. if you are sitting in room A or B1, B2, B3 etc. if you are sitting in room B. You will not be told who these people are neither during nor after the game.

This game is played in groups. You have been linked with 3 other persons and they are sitting in a different room from yours. Imagine that these three persons are related to you. One is a member of your close family (your brother or your sister for example), one is a member of your extended family (your cousin, for example), and last one is your neighbor. You may think of it as your social network.

In the game you will have make some decisions. Your decisions have economic implications in the sense that you can make some earnings or not. You will take home with you the money you have at the end of the game. During the experiment we shall not speak of Shilling, but of Experimental Currency Units (ECU). Your entire earnings will be calculated in ECU. At the end of the experiment the total amount of ECUs you have earned will be converted to Schillings at the rate of **1000 ECU = 1000 Shilling** and will be immediately paid to you in cash.

Your income is 2000 ECU. These are yours and your can exchange it for shillings and take it home at the end of the game. The different stages of the games are summarized as follows.

Before beginning the game we kindly ask you to fill the questionnaire that we provided to you. Once you have finished please put it on the side.

**1. Private decisions of your family and neighbors**

In a different room from yours the three members of social network (close family, extended family and neighbor) are given some money (5000 ECU each) that they will divide between a saving box and an investment box. How to divide the money between the two boxes is their decision. They will make this decision individually. The money that they put in the investment box can be used to generate more money. The money can also be lost. So if the investment is successful they will get back more money that they did put in the investment box. If the investment fails they loose all the money they put it in the investment box. The money that they put in the saving box just sits there. It does not generate more money. They do not loose either.

How do we determine the result of their investment? After that they divide their 5000 ECU between the saving box and the investment box, we will ask them to toss a coin. If the coin lands with **head facing up** they will be paid three times the amount of money that they put in the investment box. If the coin lands with **tail facing up** they loose the amount they put in the investment box.

So, for example, if they decide to put 3000 ECU in the investment box how much will they get? They will toss the coin and determine it. If the coin lands with head facing up they will get 3x3000 ECU = 9000 ECU. If the coin land with tail facing up they get 0. They can put as much or as little they want in the investment cup. If they like they can put everything (all the 5000 ECU) or nothing (0 ECU) in the investment box. If they like they can put everything (all the 5000 ECU) or nothing (0 ECU) in the saving box.

**2. Dissemination stage**

A member of the team will collect the information about the money that your close family, extended family and neighbors have. Your income is 2000 ECU. Your extended family, close family and neighbors know that the amount of money they have.

First example, if they decided to put in the investment box 1000 ECU and gained and 4000 ECU in the saving box. Their income is 6000 ECU. You are related to them as close family, extended family or neighbors. Your will be informed about it. Second example, your cousin decided to put 4000 ECU in the investment box and gained. His total income is 13000 ECU. Again you will be informed about it. Here is a sketch of the game:

How much money there is your social network?

TOTAL ECU of your close family member = ECU the saving box + outcome from investment box

TOTAL ECU of your extended family member = ECU the saving box + outcome from investment box

TOTAL ECU of your neighbours = ECU the saving box + outcome from investment box

ALL these information are told to you. You have been given a claim sheet. One of the members of to game team will write down the money that your close family, extended family and neighbors have.

**3. Claims stage**

You know have full information of the amount of money of your close family, your extended family, and your neighbor. Your income is 2000 ECU. At this stage of the game you can make a decision. If you wish you can make a claim to your network. You may ask some of their money. The amount of income you may ask depends upon the relationship you have. If is your close family (your brother or your sister for instance) you may ask up to 50% of your money. If is your extended family may ask up to 30% of their money. If is your neighbors may ask up to 20% of their money. How much you want to claim is your decision. You can claim 0 if you want. Let us illustrate this stage of the game with an example. Imagine you are B3. Your close family member had initially 5000 ECU and divide it in the following way: 3000 ECU in the saving box and 2000 in the investment box. Your close family member (your brother or sister) was successful in the investment. So he/she now has 3000 ECU + 3x2000 ECU = 9000 ECU. Do you want to claim some of his/her money? If yes, how much (up to a maximum of 50% of it – 4500 ECU). Your extended family member (your cousin, for instance) had initially 5000 ECU and divides it in the following way: 4000 ECU in the saving box and 1000 in the investment box. Your cousin was unsuccessful in the investment. So she now has lost 1000 ECU. Her total amount of money is therefore 4000 ECU. Do you want to claim some of it? If yes, how much (up to a maximum of 30% of it – 1200 ECU). To complete your social network lets turn now to your neighbor. Your neighbor as well had initially 5000 ECU. He divides it in the following way: 5000 ECU in the saving box and 0 in the investment box. Her total amount of money is therefore still the initial 5000 ECU. Do you want to claim some of it? If yes, how much (up to a maximum of 20% of it – 1000 ECU). Got it? Raise your hand if not clear. We will explain it again to you.

How is this information brought to you in the game? We gave you a claim sheet. A member of the game team will write down the money that your close family, extended family, and neighbor do have. You can simply write down next to these figures how much you claim. Note that you can claim any amount between 0 and the maximum allowed in the relationship.

**4. Final pay off stage**

At this stage of the game your network members know how much money you have (2000 ECU) and how much money you are claiming from them. They will decide how much money they will send you in response to your claims. This is entirely their decision. They can send you back the full amount you requested, a part of it or zero. How much in total you obtain (on top of your 2000 ECU) is for you to take home. So at the end of the game you will take home:

+2000 ECU

+ ECU given by your close family to respond to your claim

+ ECU given by your extended family to respond to your claim

+ ECU given by your neighbor to respond to your claim

**Thank you for playing – you can leave the room.**

**Payments will be done in private.** Questions?

**In summary, in the game you have three decisions to be taken.**

1. How much of the ECU of your close family you want to claim?
2. How much of the ECU of your extended family you want to claim?
3. How much of the ECU of your close family you want to claim?

**Do you have any questions? Please raise your hand; a member of the research team will come to you and answer your question in private.**

**Room 6**

Thank you for taking part in this economic game. It is important that you take your time to understand the instructions. The instructions which are distributed to you are for your private information. **Please do not communicate with the other participants during the game. This is like an exam!** Should you have any questions please raise your hand. The remainder of these instructions will explain exactly how this game is run. Since in the game you will have to make a private decision, we ask that you not tell anyone your decision either during, or after, the game. To keep anonymity you have been assigned a game ID. For instance A1, A2, A3 etc. if you are sitting in room A or B1, B2, B3 etc. if you are sitting in room B. You will not be told who these people are neither during nor after the game.

This game is played in groups. You have been linked with 3 other persons and they are sitting in a different room from yours. Imagine that these three persons are related to you. One is a member of your close family (your brother or your sister for example), one is a member of your extended family (your cousin, for example), and last one is your neighbor. You may think of it as your social network.

In the game you will have make some decisions. Your decisions have economic implications in the sense that you can make some earnings or not. You will take home with you the money you have at the end of the game. During the experiment we shall not speak of Shilling, but of Experimental Currency Units (ECU). Your entire earnings will be calculated in ECU. At the end of the experiment the total amount of ECUs you have earned will be converted to Schillings at the rate of **1000 ECU = 1000 Shilling** and will be immediately paid to you in cash.

Your income is 2000 ECU. These are yours and your can exchange it for shillings and take it home at the end of the game. The different stages of the games are summarized as follows.

Before beginning the game we kindly ask you to fill the questionnaire that we provided to you. Once you have finished please put it on the side.

**1. Private decisions of your family and neighbors**

In a different room from yours the three members of social network (close family, extended family and neighbor) are given some money (5000 ECU each) that they will divide between a saving box and an investment box. How to divide the money between the two boxes is their decision. They will make this decision individually. The money that they put in the investment box can be used to generate more money. The money can also be lost. So if the investment is successful they will get back more money that they did put in the investment box. If the investment fails they loose all the money they put it in the investment box. The money that they put in the saving box just sits there. It does not generate more money. They do not loose either.

How do we determine the result of their investment? After that they divide their 5000 ECU between the saving box and the investment box, we will ask them to toss a coin. If the coin lands with **head facing up** they will be paid three times the amount of money that they put in the investment box. If the coin lands with **tail facing up** they loose the amount they put in the investment box.

So, for example, if they decide to put 3000 ECU in the investment box how much will they get? They will toss the coin and determine it. If the coin lands with head facing up they will get 3x3000 ECU = 9000 ECU. If the coin land with tail facing up they get 0. They can put as much or as little they want in the investment cup. If they like they can put everything (all the 5000 ECU) or nothing (0 ECU) in the investment box. If they like they can put everything (all the 5000 ECU) or nothing (0 ECU) in the saving box.

**2. Dissemination stage**

A member of the team will collect the information about the money that your close family, extended family and neighbors have. Your income is 2000 ECU. Your extended family, close family and neighbors know that the amount of money you have.

First example, if they decided to put in the investment box 1000 ECU and gained and 4000 ECU in the saving box. Their income is 6000 ECU. You are related to them as close family, extended family or neighbors. Your will be informed about it. Second example, your cousin decided to put 4000 ECU in the investment box and gained. His total income is 13000 ECU. Again you will be informed about it.

They are given the opportunity, however, to pay a fee and avoid to inform you about their true total amount of money. They have the choice to pay a fee in order to inform you that they have 5000 ECU in the saving box and nothing more. First example, if they decided to put in the investment box 2000 ECU and gained and 3000 ECU in the saving box. Their income is 3x2000 ECU+ 3000 ECU. In total they will have 9000 ECU. They paid a fee and informed your network that their income is instead 5000 ECU. Second example, they decided to put 4000 ECU in the investment box and gained. their total income is 13000 ECU. They paid a fee and informed the close family, the extended family and the neighbor that you have only 5000 ECU in the saving box. Third example, they have divided their 5000 ECU in the following way. 2000 ECU in the investment box and 3000 ECU in the saving box. They toss the coin and lost (tail facing up). Their final income is 3000 ECU. You will be informed that they have 3000 ECU.

To recap: they have to choose how to allocate your 5000 ECU between the saving box and the investment box and if you want to avoid to inform the others.

How much is the fee? The fee is 500 if they want to hide from your close family your income, 300 ECU if they want to hide from your extended family your income, 200 ECU if they want to conceal the amount from your neighbor. They can decide not to pay the fees and disclose the full amount in the saving box and in the investment box. They can decide to pay the fees only for some members of the network and not other. For instance, if they do not want to disclose your full amount to their cousins and neighbor they will pay 500 ECU. Or if they do not want to disclose the full amount to their neighbor, but are happy with full disclosure to your family (close and extended) they pay a fee of 200 ECU. It is entirely up to them. It is their decision in the game. Got it? If not clear raise your hand now. Here is it a sketch of this stage of the game.

How much money there is your social network?

TOTAL ECU of your close family member = ECU the saving box + outcome from investment box – fees in any

TOTAL ECU of your extended family member = ECU the saving box + outcome from investment box– fees in any

TOTAL ECU of your neighbours = ECU the saving box + outcome from investment box– fees in any

You have been given a claim sheet. One of the members of to game team will write down the money that your close family, extended family and neighbors have.

**3. Claims stage**

You know have information of the amount of money of your close family, your extended family, and your neighbor. Your income is 2000 ECU. At this stage of the game you can make a decision. If you wish you can make a claim to your network. You may ask some of their money. The amount of income you may ask depends upon the relationship you have. If is your close family (your brother or your sister for instance) you may ask up to 50% of your money. If is your extended family may ask up to 30% of their money. If is your neighbors may ask up to 20% of their money. How much you want to claim is your decision. You can claim 0 if you want. Let us illustrate this stage of the game with an example. Imagine you are B3. Your close family member had initially 5000 ECU and divide it in the following way: 3000 ECU in the saving box and 2000 in the investment box. Your close family member (your brother or sister) was successful in the investment. So he/she now has 3000 ECU + 3x2000 ECU = 9000 ECU. Do you want to claim some of his/her money? If yes, how much (up to a maximum of 50% of it – 4500 ECU). Your extended family member (your cousin, for instance) had initially 5000 ECU and divides it in the following way: 4000 ECU in the saving box and 1000 in the investment box. Your cousin was unsuccessful in the investment. So she now has lost 1000 ECU. Her total amount of money is therefore 4000 ECU. Do you want to claim some of it? If yes, how much (up to a maximum of 30% of it – 1200 ECU). To complete your social network lets turn now to your neighbor. Your neighbor as well had initially 5000 ECU. He divides it in the following way: 5000 ECU in the saving box and 0 in the investment box. Her total amount of money is therefore still the initial 5000 ECU. Do you want to claim some of it? If yes, how much (up to a maximum of 20% of it – 1000 ECU). Got it? Raise your hand if not clear. We will explain it again to you.

How is this information brought to you in the game? We gave you a claim sheet. A member of the game team will write down the money that your close family, extended family, and neighbor do have. You can simply write down next to these figures how much you claim. Note that you can claim any amount between 0 and the maximum allowed in the relationship.

**4. Final pay off stage**

At this stage of the game your network members know how much money you have (2000 ECU) and how much money you are claiming from them. They will decide how much money they will send you in response to your claims. This is entirely their decision. They can send you back the full amount you requested, a part of it or zero. How much in total you obtain (on top of your 2000 ECU) is for you to take home. So at the end of the game you will take home:

+2000 ECU

+ ECU given by your close family to respond to your claim

+ ECU given by your extended family to respond to your claim

+ ECU given by your neighbor to respond to your claim

**Thank you for playing – you can leave the room.**

**Payments will be done in private.** Questions?

**In summary, in the game you have three decisions to be taken.**

1. How much of the ECU of your close family you want to claim?
2. How much of the ECU of your extended family you want to claim?
3. How much of the ECU of your close family you want to claim?

**Do you have any questions? Please raise your hand; a member of the research team will come to you and answer your question in private.**

**Risk preference experiment**

Bad harvest

(Head)

200
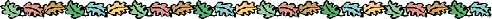


Good harvest

(Tail)

3600

Bad harvest

(Head)

1000
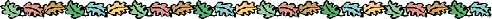


Good harvest

(Tail)

1000

Bad harvest

(Head)

800
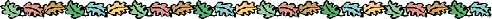


Good harvest

(Tail)

2400

Bad harvest

(Head)

900
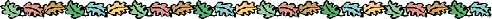


Good harvest

(Tail)

1800

Bad harvest

(Head)

600
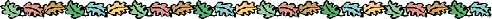


Good harvest

(Tail)

3000

Bad harvest

(Head)

0
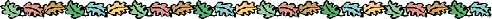


Good harvest

(Tail)

4000

Plot 1.___

Plot 2.___

Plot 3.___

Plot 4.___

Plot 5.___

Plot 6.___

The respondent is asked to choose between the different farming plots (plot 1 to plot 6); Each plot gives either the bad harvest yield or a good harvest yield. For instance, plot 2 gives 900 Shillings if the season is bad (bad harvest), but it gives 1800 Shillings if the season is good (good harvest). Then, a coin will be tossed - head represent bad harvest and tail represent good harvest - the result will be recorded and the amount corresponding to the choice and the coin toss will be paid out to the respondent)

*Extension officer, please read the following:* Imagine you can select 1 of 6 plots. On plot one, you earn 1000 Tsh if the season is bad and also 1000 Tsh if the
